# Enhanced stability of filament-type resistive switching by interface engineering

**DOI:** 10.1038/srep43664

**Published:** 2017-05-02

**Authors:** Y. B. Zhu, K. Zheng, X. Wu, L. K. Ang

**Affiliations:** 1 SUTD-MIT International Design Center (IDC), Singapore University of Technology and Design (SUTD), 8 Somapah road, 487372, Singapore; 2 Shanghai Key Laboratory of Multidimensional Information Processing, Department of Electrical Engineering, East China Normal University, Shanghai, 200241, China

## Abstract

The uncontrollable rupture of the filament accompanied with joule heating deteriorates the resistive switching devices performance, especially on endurance and uniformity. To suppress the undesirable filaments rupture, this work presents an interface engineering methodology by inducing a thin layer of NiO_x_ into a sandwiched Al/TaO_x_/ITO resistive switching device. The NiO_x_/TaO_x_ interface barrier can confine the formation and rupture of filaments throughout the entire bulk structure under critical bias setups. The physical mechanism behind is the space-charge-limited conduction dominates in the SET process, while the Schottky emission dominates under the reverse bias.

The modern non-volatile memory has been successfully scaled down to achieve ultra-high-density storage with the development of photolithography technology. However, the conventional charge storage memory is approaching the technical limit and three-dimensional (3D) crossbar structure would be a promising scaling scheme for next generation non-volatile memory^1^,^2^. Resistive switching random access memory (RRAM) is favorable with its high-density 3D crossbar integration ability, fast operation speed and low power consumption^3^,^4^. In the last two decades, oxide based RRAM has been intensively studied^5^,^6^, and TaO_x_ has been recognized as one of potential choices due to its excellent endurance and retention performance^7^,^8^. It is well acknowledged that there are only two stable stoichiometric solid phases in TaO_x_: (a) high oxygen defective phase (or conductive path) and (b) low oxygen defective phase (or insulating matrix)^9^,^10^. Such property guarantees relatively stable filaments formation in the TaO_x_ based RRAM devices^11^,^12^.

However, nearly all the filaments-type RRAM could not avoid a critical issue that the RESET of device will involve the rupture of filaments, either by ion migration or Joule heating^13^,^14^. This issue inevitably causes some negative effects, such as scattered switching voltage distribution and endurance deterioration^15^,^16^. Recently, some groups have proposed different designs to improve the filaments property in TaO_x_ based RRAM. For example, Lee *et. al*. has presented a so-called Metal-Insulator-Base-Metal (MIBM) structure to constrain the filaments in a thin highly insulating Ta_2_O_5-x_ layer and to use another less insulating TaO_2-x_ layer as the reservoir of oxygen vacancies to enhance the filaments transition in the insulator layer^17^. By applying different voltages on the 1-transistor-1-memoristor (1T1M) device, Miao *et al*. realized the manipulation of the filaments in TaO_x_ which related to the oxygen vacancies, and obtained varying switching behavior^18^. However, these designs still involve the destruction of filaments at the interface area during the reset process, which infers to the abrupt decrease of current by several orders in the *I-V* characteristic^19^,^20^. In this paper, we introduce a thin NiO_x_ layer between TaO_x_ and top electrode to introduce a barrier, which successively prevents the abrupt rupture of the filaments in the RESET process. Our proposal in using a hybrid structure of n-type TaO_x_ and p-type NiO_x_ interface engineering to control the formation of the filaments, instead of breaking and reconnecting them. Our design effectively improves the endurance and uniformity of resistive switching (RS) behavior, and offers an alternative design for future RRAM device.

## Results

**Figure 1:**
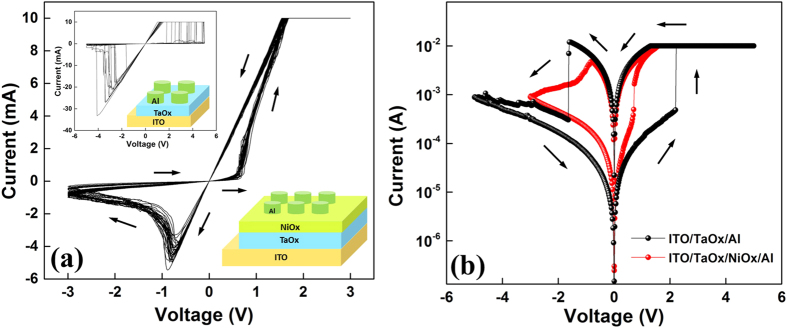
(**a**) The bipolar RS cycles and structure schematic of the ITO/TaO_x_/NiO_x_/Al device, the inset shows the switching cycles and structure schematic of device without the NiO_x_ layer; (**b**) Comparison of the switching cycles between the ITO/TaO_x_/NiO_x_/Al and the ITO/TaO_x_/Al devices in *log(I)-V* scale.

The bipolar RS cycles of ITO/TaO_x_/NiO_x_/Al device is demonstrated in Fig. 1a. The electroforming procedure is needed to initialize the resistive switching process (not shown here). As the arrow indicates, the device is initially at the high resistance state (HRS) and the switching cycle starts with a positive DC sweep. A dramatic current increment occurs at around 0.7 V, which corresponds to the SET operation, and the device reaches the low resistance state (LRS). A compliance current (CC) of 10 mA is imposed in order to prevent the permanent breakdown of oxide layers at >1.5 V. As the bias sweeps back from positive to negative region, the device maintains the linear I-V characteristics from 1.5 to ~−0.8 V. The device is reset to HRS again and this completes one switching cycle. The same multiple switching cycles of the regular ITO/TaO_x_/Al device (without the added NiO_x_ barrier) is also plotted in the inset of Fig. 1a. Several apparent distinctions could be noticed through the comparison. The distribution of the switching threshold voltage is more concentrated and the switching curves are more uniform for the device with the inserted NiO_x_ layer. The two batch of switching curves having different shapes could be further distinguished in the log-log I-V plotting as shown in Fig. 1b. The LRS behaviors of both two devices are similar, however, an obvious abrupt change of current in both SET and RESET processes could be observed only in the regular ITO/TaO_x_/Al device, but not in our newly proposed ITO/TaO_x_/NiO_x_/Al design. The transition of the high and low resistance states becomes smoother after the introduction of NiO_x_ layer between TaO_x_ and top electrode, which indicates that two different switching mechanisms are present in these two different devices (see below).

**Figure 2:**
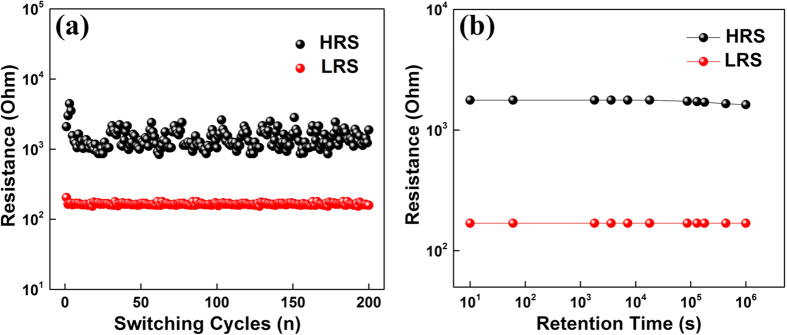
(**a**) Endurance performance of the ITO/TaO_x_/NiO_x_/Al RRAM; (**b**) Retention performance of the ITO/TaO_x_/NiO_x_/Al RRAM.

The HRS and LRS resistance of ITO/TaO_x_/NiO_x_/Al device at 200 DC switching cycles is shown in Fig. 2a in order to characterize the endurance performance. The LRS resistance shows excellent consistency and the HRS resistance also becomes relatively stable after first several cycles. The resistance window is maintained well at about one order of magnitude difference, which is sufficient to realize binary storage. The retention characteristic at room temperature is shown Fig. 2b. After continuous sampling for around 10^6^ s, the LRS resistance remains unchanged while the HRS resistance shows a little bit of decay after 10^5^ s. These results manifest the potential application of non-volatile memory with such proposed device structure.

**Figure 3:**
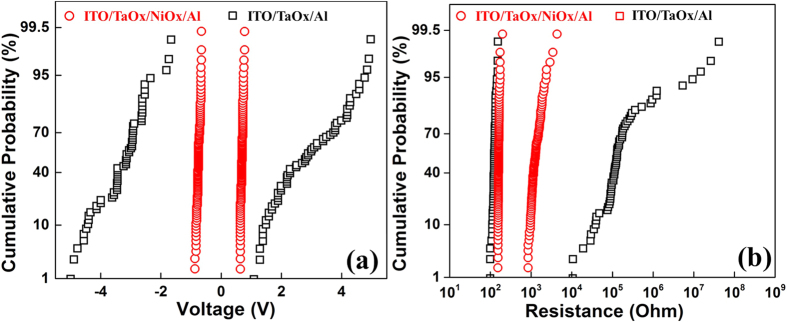
Cumulative probability comparison of (**a**) SET & RESET voltages, and (**b**) LRS & HRS resistances between the ITO/TaO_x_/NiO_x_/Al and the ITO/TaO_x_/Al devices.

To further reveal the improvement by inserting a thin NiO_x_ layer into the ITO/TaO_x_/Al structure, the cumulative distribution of switching voltages and two states resistance are also analyzed. In Fig. 3a, both SET and RESET voltages of ITO/TaO_x_/NiO_x_/Al device are highly centralized, which are respectively ranged from 0.64 to 0.78, and −0.68 to −0.88 volts, while the voltage distribution of ITO/TaO_x_/Al one is much scattered (1.3to 4.9 volts, −1.7 to −5.0 volts), which is consistent with the feature shown in Fig. 1. In Fig. 3b, the distributions of the LRS resistance of two devices are both in narrow range (100 Ω to 200 Ω); however, the distribution of the HRS resistance is greatly optimized in the ITO/TaO_x_/NiO_x_/Al structure (from a big range of 10 KΩ–100 MΩ to a smaller range of 1–4KΩ). Moreover, the ITO/TaO_x_/Al device only exhibits less than 100 stable switching cycles at our DC measurement, and the endurance has been improved with additional NiO_x_ layer as indicated in Fig. 2a.

## Analysis and Discussions

**Figure 4:**
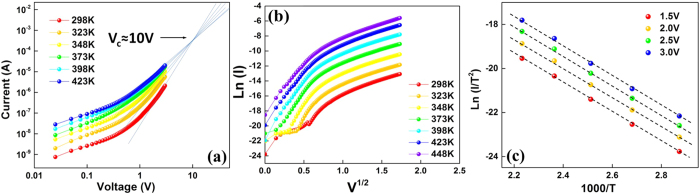
*I-V* characterization before electroforming process: (**a**) *Log(I)-V* plotting under the positive bias; (**b**) *Ln (I*) − *V*^*1*/*2*^ plotting under the negative bias; (**c**) Arrhenius plots of *Ln (I*/*T*^*2*^) − *1*/*T* under the negative bias.

To determine the mechanism for such a remarkable improvement by introducing a thin p-type NiO_x_ layer, the conduction properties of the as-prepared device and the RS processes are studied. Before electroforming operation, the as-prepared ITO/TaO_x_/NiO_x_/Al stacks are in the high resistance state. At this state, the *I-V* curves with increasing temperatures were plotted in log-log scale in Fig. 4a and b, and we can observe that the cases in the positive and negative sweep ranges are totally different. When the positive bias is applied on top electrode, the IV curves all have a slope of around one at low voltage region (ohmic conduction), but gradually converge to *V*_*c*_ as the electric field increases as demonstrated in Fig. 4a. This is the typical behavior of the trap-limited-conduction^21^,^22^. We consider the energy state of the traps solid is described by an exponential function *N(E*) = *N*_*t*_/(*kT*_*t*_)exp[(*E* − *E*_*c*_)/*kT*_*t*_], where *N*_*t*_ is the trap density, *E*_*c*_ is the band edge energy, *k* is Boltzmann’s constant and *T*_*t*_ is the characteristic trap temperature. This regime is known as the trap-limited SCLC or Mark-Helfrich (MH) law^23^ with its current density given by:


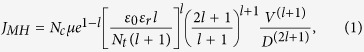


where *N*_*c*_ is the effective density of states corresponding to the energy at the bottom of the conduction band, *μ* is the electron mobility, and *1* = *T*_*t*_/*T* is the ratio of distribution of traps to the free carriers. The traps will be gradually filled as the electric field increased at all temperatures. When the applied voltage reach a critical value, all traps will be filled. This critical voltage is independent of temperature and given by ref. 22:


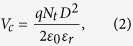


where *q* is the electron charge. By extrapolating the *I-V* curves, we obtain a typical value for *V*_*c*_, which is about 10 volts for our device. The electron transport behavior is confirmed to be in the trap-limited region.

When negative bias is applied, the IV curves follow the Schottky emission^24^:


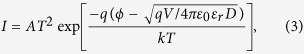


where 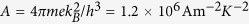 is the Richardson constant, *T* is the temperature, *ϕ* is the barrier height. By re-plotting the *I-V* curves in the form of 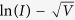 (see Fig. 4b), the linear and parallel feature at high (negative) voltage region agrees well with the Schottky emission process. Furthermore, the Arrhenius plots in Fig. 4c show the linearity of 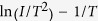 relationship, which also confirms the dominant conduction in the negative voltage region is Schottky emission^25^. For comparison, we also analyzed the *I-V* characteristics of Al/NiO_x_/Al and ITO/TaO_x_/ITO stacks. It is found that the SCLC is the main conduction mechanism in these two stacks, which excludes the barrier effect at Al/NiO_x_ and ITO/TaO_x_ interfaces. Thus, we conclude the Schottky emission observed in ITO/TaO_x_/NiO_x_/Al is attributed to the n-type TaO_x_ and p-type NiO_x_ interface barrier.

**Figure 5:**
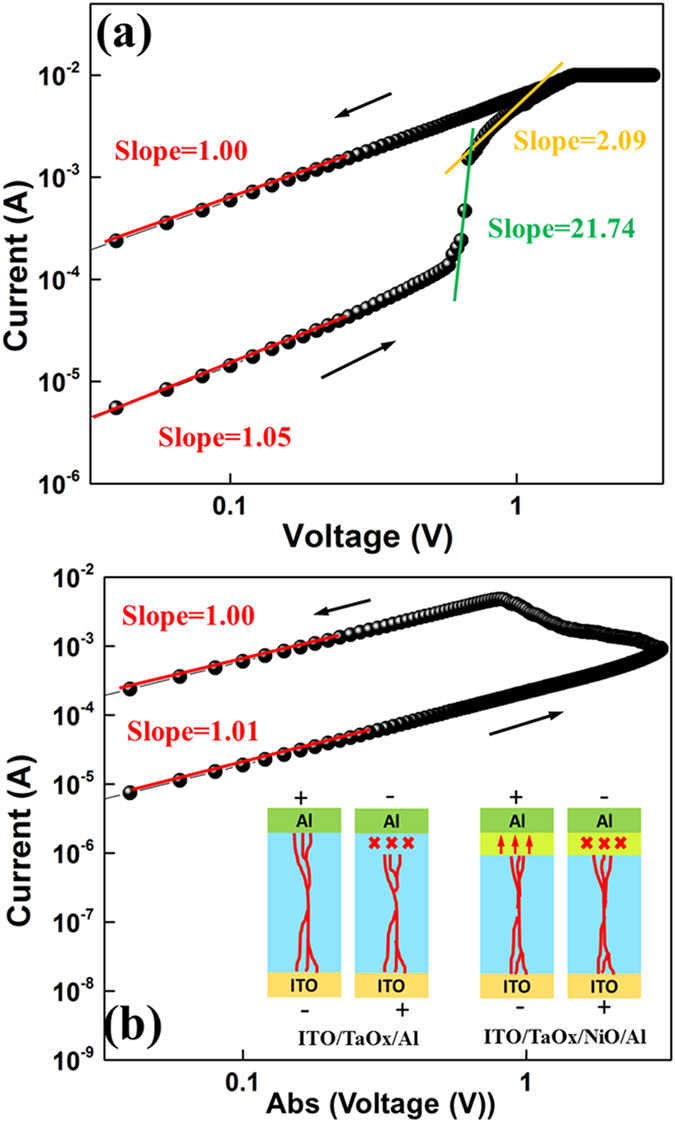
Log-log scale *I-V* characterization after electroforming process: (**a**) SET under the positive bias; (**b**) RESET under the negative bias.

The conduction properties in LRS and HRS are also investigated. From the I-V curves plotted in the log-log scale in Fig. 5a, a typical SCLC behavior in HRS could be identified. As the injected carriers are not comparable with intrinsic thermal carriers in low voltage region, ohmic conduction with slope of about 1 ( = 1.05 indicated in Fig. 5a) is expected. The current density depends proportionally on the concentration of thermally generated carriers *n*_0_, given as


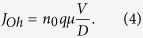


With higher injecting current density, the injected carriers promptly fill the traps in the oxides, which lead to a high current gain with a slope of 21.74. These traps are well known to be as oxygen vacancies for n-type TaO_x_ and Ni vacancies for p-type NiO_x_. This region is known as the trap-limited SCLC or Mark-Helfrich (MH) law^23^ given by Eq. (1) with slop *1* + 1. When the traps are gradually occupied fully, the slope reduces to around 2 ( = 2.09 indicated in Fig. 5a), indicating that the conduction enters the trap-free SCLC, and the current density is determined by the Mott-Gurney (MG) law^26^,^27^:


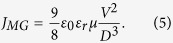


For the LRS, the high current with ohmic conduction is the standard metallic property of filament formed in oxide^28^. In the RESET process as shown in Fig. 5b, the ohmic behavior with the slope of 1 is still maintained in LRS until the filament based conduction is gradually impeded and the device gets back to HRS. The slope of 1 in HRS corresponds to the low thermal current in oxide again.

**Figure 6:**
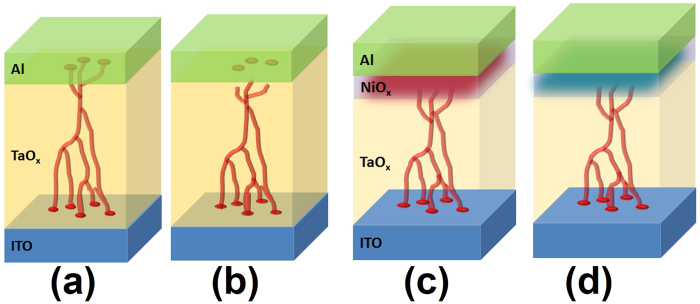
Schematics of the resistive switching mechanism comparison between (**a**,**b**) the ITO/TaO_x_/Al, and (**c**,**d**) the ITO/TaO_x_/NiO_x_/Al RRAM devices.

Based on the above results, the different RS process in these two stacks could be described by taking into the consideration of both filaments and interface barriers effects (see Fig. 6). For a ITO/TaO_x_/NiO_x_/Al device (Fig. 6c), the positive voltage on top electrode will reduce the TaO_x_/NiO_x_ barrier and enables injected electron hopping among the traps (usually oxygen vacancies in TaO_x_) to realize the conduction throughout the whole stacks^29^. When the voltage increase, we enter the SCLC regime, and the oxygen vacancies migrate along the applied field (to the bottom ITO/TaO_x_ interface), which will trigger soft dielectric breakdown from bottom up. When the filaments formed completely with the migration of oxygen vacancies and successively passed through the n-TaO_x_/p-NiO_x_ interface to the top electrode, the device is set to LRS states. Concurrently, the oxygen vacancies in NiO_x_ will be extracted to TaO_x_ with the positive bias, which will also make both oxide layers to be more conductive to facilitate the filament based conduction. Thus the LRS is sustainable even after removing the external field due to the existence of filament, which satisfies the non-volatile storage.

Subsequently, when negative voltage is applied on the top electrode (Fig. 6d), the filamentary conduction still maintains in a small region. However, the increasing reverse bias on n-TaO_x_/p-NiO_x_ junction will raise the barrier height and hinder the conduction to reset the device to HRS. Similarly, the negative voltage will also absorb some oxygen vacancies from TaO_x_ back into NiO_x_ and make them to be highly resistive, further impeding the reverse current. It is worth to mention that the filaments in TaO_x_ are not broken here, because the current is reducing gently instead of abrupt drops as shown in Fig. 1b. This provides the evident that the gradually rising TaO_x_/NiO_x_ barrier weakens the filamentary conduction through the interface, avoiding the rupture of filaments. Therefore, the next SET cycle will only need to lower the barrier and enable the filaments passing through again without the requirement of reconnecting or repairing any rupture filaments.

For the traditional ITO/TaO_x_/Al device (Fig. 6a and b), a filament rupture procedure at TaO_x_/ITO interface with negative bias is required in RESET operation, which is consistent to a sudden current drop of several orders depicted in Fig. 1b 30. From the comparison, we could understand that the improvement of RS by inserting a NiO_x_ layer is mainly ascribed to employing an interface barrier effect to control the filaments conduction without making any destructive operation on the filaments as in the RS processes. The resistance fluctuation, especially in the HRS, is greatly suppressed because the HRS originates from the reverse bias of barrier, not from the randomly broken filaments. The switching threshold value is also optimized because tuning the barrier height is much more stable than the ruinous handling of filaments. At the same time, a better endurance could be also obtained by selecting this gentle RESET strategy. The major defect in NiOx is Ni vacancy, so under the reverse bias, the oxygen vacancies migrate into NiOx, counteracting the Ni vacancies and form more stoichiometric NiO which are higher resistive. The process is similar like the formation of space charge region in PN junction, which is due to the major carrier election in N region diffusing into P region. Such process somehow causes some so-called “damage” on the filament but only at the TaOx/NiOx interface region. Such “damage” is actually a part of the “barrier tuning filament” effect, and will not affect the whole filament establish inside TaOx.

### Summary

In conclusion, an interface engineering resistive switching device of ITO/TaO_x_/NiO_x_/Al structure was designed and fabricated. The device shows an enhanced improvement in the endurance performance and the distribution of switching voltages and resistance over the traditional ITO/TaO_x_/Al structure. For the as-prepared device, SCLC is the main conduction mechanism in the positive bias regime, and Schottky emission dominates the conduction in negative bias regime, which proves the formation of a confined TaO_x_/NiO_x_ interface barrier. The introduction of such an interface barrier with TaO_x_ can control and manipulate the passing status of filaments efficiently. The proposed design avoids the rupture of filaments in RESET process, which will enable a more uniform and stable resistive switching behavior comparable to other RRAM devices^12^,^17^,^31^.

### Experiment Section

The Al/NiO_x_/TaO_x_/ITO RRAM devices were fabricated by magnetron sputtering system at room temperature. The ITO commercial glass was employed as the substrate for deposition and bottom electrode. A 50 nm n-type TaO_x_ layer was deposited by using a Ta_2_O_5_ target with the RF sputtering power of 100 W. The pressure sputtering chamber was maintained at an Ar (Argon) atmosphere of 5 × 10^−3^ Torr. A 10 nm p-type NiO_x_ layer followed in the same chamber atmosphere by using a NiO target with the RF power of 70 W. Lastly, a 200 nm Al top electrode layer was deposited with a shadow mask by using DC sputtering power of 60 W. A traditional ITO/TaO_x_/Al RRAM device was also fabricated for comparison. The conduction type of TaO_x_ and NiO_x_ were confirmed by Hall effect measurement. *I-V* characterization was carried out on a probe station with the Keithley 4200 SCS semiconductor parameter analyzer. During the DC mode measurement, the biases with different polarities were applied on the Al top electrode and the ITO bottom electrode was always grounded.

## Additional Information

**How to cite this article:** Zhu, Y. B. *et al*. Enhanced stability of filament-type resistive switching by interface engineering. *Sci. Rep.* **7**, 43664; doi: 10.1038/srep43664 (2017).

**Publisher's note:** Springer Nature remains neutral with regard to jurisdictional claims in published maps and institutional affiliations.
